# Insights Into Nitric Oxide Modulated Quorum Sensing Pathways

**DOI:** 10.3389/fmicb.2019.02174

**Published:** 2019-09-24

**Authors:** Ilana Heckler, Elizabeth M. Boon

**Affiliations:** Department of Chemistry, Institute of Chemical Biology & Drug Discovery, Stony Brook University, Stony Brook, NY, United States

**Keywords:** nitric oxide, quorum sensing, gas sensing, hemoproteins, virulence, biofilm

## Abstract

The emerging threat of drug resistant bacteria has prompted the investigation into bacterial signaling pathways responsible for pathogenesis. One such mechanism by which bacteria regulate their physiology during infection of a host is through a process known as quorum sensing (QS). Bacteria use QS to regulate community-wide gene expression in response to changes in population density. In order to sense these changes in population density, bacteria produce, secrete and detect small molecules called autoinducers. The most common signals detected by Gram-negative and Gram-positive bacteria are acylated homoserine lactones and autoinducing peptides (AIPs), respectively. However, increasing evidence has supported a role for the small molecule nitric oxide (NO) in influencing QS-mediated group behaviors like bioluminescence, biofilm production, and virulence. In this review, we discuss three bacteria that have an established role for NO in influencing bacterial physiology through QS circuits. In two *Vibrio* species, NO has been shown to affect QS pathways upon coordination of hemoprotein sensors. Further, NO has been demonstrated to serve a protective role against staphylococcal pneumonia through S-nitrosylation of a QS regulator of virulence.

## Introduction

Quorum sensing (QS) refers to the process by which bacteria regulate gene expression in response to small molecules called autoinducers. Autoinducers, typically acylated homoserine lactone molecules in Gram-negative bacteria or oligopeptides in Gram-positive bacteria, are synthesized inside the cell and freely diffuse across the membrane. As cell density rises, the concentration of autoinducers in the surrounding environment also increases. When a threshold level of autoinducers is reached, autoinducers bind to their respective receptors and initiate a signal transduction cascade downstream, ultimately resulting in the repression or expression of specific genes. QS has been shown to mediate several bacterial processes including bioluminescence, sporulation, competence, antibiotic production, biofilm formation, and virulence factor production (Miller and Bassler, 2001).

Bacterial receptors of autoinducers can be membrane bound hybrid histidine kinase proteins (Ng and Bassler, 2009). Hybrid histidine kinases contain a receiver domain, in addition to an input domain and kinase core. Upon binding ATP, the receptor will autophosphorylate on a conserved histidine residue in the kinase core. Phosphate is then transferred from the histidine residue to an aspartate residue on the same polypeptide. Hybrid histidine kinases rely on an intermediate histidine phosphotransferase (HPT) protein to then transfer the phosphate from the receiver domain of the kinase, to the receiver domain of a response regulator. The most common autoinducers sensed by Gram-negative bacteria are acylated homoserine lactones, or an interfuranosyl borate diester interspecies signal named autoinducer-2 (Papenfort and Bassler, 2016). In Gram-positive bacteria, autoinducing peptides (AIPs) which are synthesized by ribosomes and secreted extracellularly by specific transporters, bind to receptor kinases at high cell density to initiate QS pathways.

Nitric oxide (NO) is a diatomic membrane-permeable gas molecule that has been implicated in a wide range of physiological processes in both eukaryotes and bacteria. Many of the interesting biological properties of NO can be attributed to its unique physical properties. Due to its size, neutral charge, and hydrophobic nature, NO is able to freely diffuse into the cytosol, where it may undergo a variety of reactions including protein S-nitrosylation and metal coordination. While at high (micromolar) concentrations, NO is cytotoxic, at low concentrations, NO has been shown to be a signaling molecule in both bacteria and eukaryotes (Murphy, 1999; Arora et al., 2015).

In mammals, NO is synthesized by nitric oxide synthases (NOS) from L-arginine, oxygen and NADPH. The first report of bacterial NOS activity came from studies of the *Nocardia* species (Chen and Rosazza, 1994). Although it does not appear that most bacteria synthesize NO enzymatically, bacteria can produce NO as a by-product of denitrification, through the anaerobic reduction of nitrite by nitrite reductase. NO is then subsequently reduced to nitrous oxide by NO reductase. Indeed, the expression of nitrite reductase and NO reductase in *Pseudomonas aeruginosa* biofilms were found to be QS-dependent, suggesting that QS is responsible for the maintenance of NO levels in this organism (Hentzer et al., 2005).

Bacteria could also respond to NO that is produced by a eukaryotic host during symbiotic or pathogenic engagement. The ability to respond to NO to mediate QS related behaviors may provide microbes with an evolutionary advantage when encountering NO during infection of a host. In addition to a role for NO as a QS signal, there may also be a connection between QS and the regulation of intracellular levels of NO.

## No Affects Quorum Sensing Pathways Through Ligation to Cytosolic Hemoprotein Receptors

Evidence has suggested that bacteria can respond to low, physiologically relevant levels of NO for the regulation of cellular processes such as biofilm formation and dispersion (Arora et al., 2015). In mammals, NO sensing is essential to regulating vasodilation and is carried out by the soluble guanylyl cyclase (sGC) receptor, which coordinates NO at the ferrous center of a heme cofactor. Homologs of the heme binding domain of sGC have been discovered in bacteria, suggesting a role for NO in regulating bacterial physiology (Iyer et al., 2003). The most well characterized NO sensor in prokaryotes is a family of hemoproteins named heme-nitric oxide/oxygen (H-NOX) proteins (Karow et al., 2004; Plate and Marletta, 2017). H-NOX proteins bind NO selectively, and with picomolar affinity (Tsai et al., 2012). H-NOX domains are commonly encoded in putative operons with cyclic di-guanosine monophosphate (c-di-GMP) processing enzymes and/or histidine kinase proteins. c-di-GMP is a bacterial secondary messenger molecule that plays a role in controlling the switch between motile and sessile/biofilm lifestyles. For example, in *Shewanella oneidensis*, NO bound H-NOX was found to inhibit the activity of an associated histidine kinase, resulting in decreased phosphorylation of a response regulator protein involved with biofilm formation through modulation of c-di-GMP levels (Plate and Marletta, 2012). Recently, NO bound H-NOX was shown to also influence bacterial biofilm formation in the marine bacterium *Vibrio fischeri* through inhibition of its associated histidine kinase, HahK (Thompson et al., 2019).

However, not all bacteria that have been shown to respond to NO encode for an H-NOX protein in their genome. This discrepancy led to the discovery of another bacterial heme bound NO sensing protein named NosP. NosP was first characterized in **P. aeruginosa** where it was found to have a role in biofilm formation (Hossain and Boon, 2017). In the human pathogen **Vibrio cholerae**, and in the marine pathogen **Vibrio harveyi**(recently reclassified as **Vibrio campbellii**) (Lin et al., 2010), NO has been implicated in mediating QS through pathways involving either H-NOX or NosP, and will be discussed here. In addition, our lab has found evidence for a similar NO responsive QS circuit in the gastroenteritis causing bacterium **Vibrio parahaemolyticus** (Ueno et al., 2019).

### *Vibrio harveyi* Biofilm, Bioluminescence, and Motility Are Regulated by a NO-Mediated QS Pathway

**FIGURE 1:**
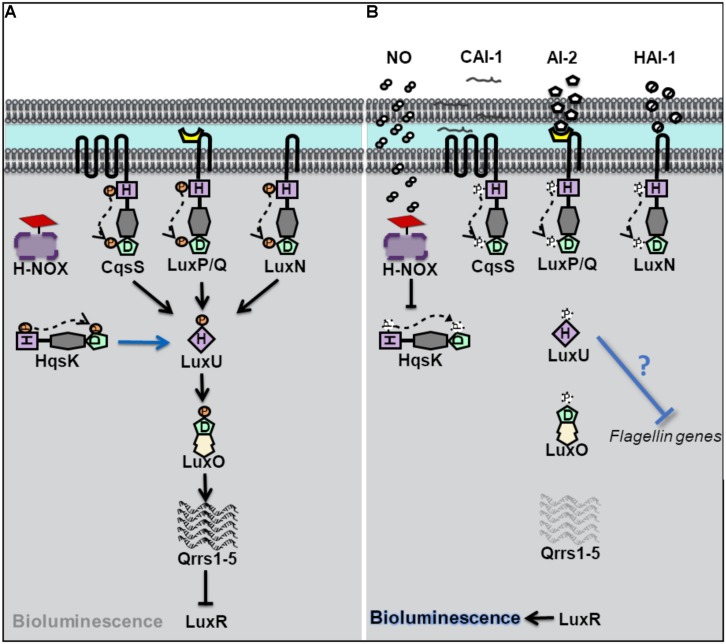
Quorum sensing (QS) in *Vibrio harveyi* at high and low cell density. **(A)** At low cell density, when autoinducer concentration is small, *V. harveyi* hybrid histidine kinase receptors CqsS, LuxPQ, and LuxN transfer phosphate to a histidine phosphotransfer protein, LuxU. In the absence of NO, the cytosolyic hybrid histidine kinase HqsK, acts as a kinase, and participates in phosphotransfer with LuxU. Phosphorylated LuxU transfers phosphate to the transcription factor LuxU, resulting in the transcription of four small regulatory RNA molecules (Qrrs 1-5). Qrrs inhibit the expression of LuxR, the transcription regulator responsible for luciferase production and activator of biofilm related genes. **(B)** QS sensing signal transduction in *V. harveyi* at high cell density and in the presence of NO. When cell density is high, and autoinducer concentrations rise, CqsS, LuxPQ, and LuxN act as phosphatases, dephosphorylating LuxU. Qrrs are not expressed and LuxR is transcribed, resulting in bioluminescence, and the activation of biofilm related genes. In the presence of NO, NO bound H-NOX inhibits the autophosphorylation of HqsK resulting in decreased phosphate flow downstream, in addition to the repression of flagellin proteins by an unknown mechanism.

*Vibrio harveyi* is a bioluminescent opportunistic marine pathogen often found in tropical waters. *V. harveyi* bioluminescence, as well as biofilm, virulence and flagellar production, is controlled by QS. *V. harveyi* is thought to respond to at least three different autoinducers through the binding of three separate QS receptors (Bassler et al., 1993, 1994; Henke and Bassler, 2004). These receptors, LuxN, LuxQ, and CsqS are transmembrane hybrid histidine kinases. LuxQ also utilizes an accessory protein, LuxP, to sense its cognate autoinducer (Bassler et al., 1994; Neiditch et al., 2006). When cell density is low, the receptors LuxN, LuxQ, and CsqS autophosphorylate and subsequently transfer phosphate to a single histidine phosphotransfer protein LuxU (Figure 1). Phosphorylated LuxU then transfers phosphate downstream to a transcription factor LuxO which promotes the transcription of Quorum Regulatory RNAs (qrrs) (Freeman and Bassler, 1999a, b). Qrrs destabilize the mRNA encoding LuxR, the transcription regulator responsible for luciferase production and activator of biofilm related genes, consequently inhibiting bioluminescence as the light producing *lux* operon is not expressed (Lenz et al., 2004). However, when cell density increases, and autoinducer concentration is high, the receptors switch from kinases to phosphatases resulting in the dephosphorylation and inactivation of LuxO, and subsequent translation of LuxR leading to light production.

*Vibrio harveyi* has also been shown to contain a NO-mediated QS pathway (Henares et al., 2012). Low cell density cultures of *V. harveyi* exhibit increased bioluminescence in the presence of exogenous nanomolar NO, suggesting that NO can modulate QS pathways. The effect on NO on QS was attribute to a fourth hybrid histidine kinase, named Hqsk, that has both kinase and phosphatase activities and is able to transfer phosphate to LuxU. Unlike the other three previously characterized *V. harveyi* kinase receptors, HqsK is cytosolic, and does not contain a sensory domain in its primary sequence. Instead, HqsK activity was found to be regulated through interaction with an NO-sensitive H-NOX protein. NO-bound *Vh*H-NOX inhibits the kinase activity of HqsK which contributes to a loss of phosphorylated LuxU and a subsequent increase in light production through the LuxU/LuxO pathway described above. It should be reiterated here that *V. harveyi* contains three additional AI receptors, which together overwhelm the effect of NO at high cell density (when, presumably, the other three AIs are present at high concentration); the addition of NO to high cell density cultures of *V. harveyi* was not found to significantly increase bioluminescence (Henares et al., 2012). This suggests that NO may influence bioluminescence only at low cell density, or perhaps be only a minor contributor to the total QS output.

In addition to bioluminescence, NO was found to influence QS regulation of biofilm and flagellar production in *V. harveyi* (Henares et al., 2013). Many bacteria, including *V. harveyi*, rely on QS to initiate a switch between a motile and sessile (or biofilm) lifestyle. At high cell density, *V. harveyi* enters biofilm through the negative regulation of genes involved with motility (Waters and Bassler, 2006). Analogous to a high-density state, the addition of low nanomolar (50 nM) NO to cultures of *V. harveyi* resulted in thicker biofilms compared to cultures grown in the absence of NO. Further, the addition of NO to a Δ*hnoX* mutant strain lost the same biofilm enhancement phenotype as the wildtype strain, indicating that *Vh*H-NOX is involved in the NO-mediated biofilm pathway. As described previously, H-NOX plays a role in *V. harveyi* QS through an interaction with HqsK. Interestingly, the addition of autoinducers to cultures of *V. harveyi* did not increase biofilm to the same degree that NO addition had (Henares et al., 2013). This finding suggests that the NO/H-NOX pathway is primary in the regulation of biofilm.

The initial stage of biofilm formation, when surface attachment occurs, is correlated with a decrease in motility and is dependent on functional flagella. While functional flagella are critical for colonization and initial attachment, late stage biofilms are often made up of bacteria that have lost their flagella. In the marine bacterium *V. fischeri*, flagellar proteins have been previously shown to be negatively regulated by QS (Lupp and Ruby, 2005). iTRAQ proteomics analysis was performed on *V. harveyi* in the presence of NO. Upon addition of 50 nM NO, the relative abundance of several *V. harveyi* flagellin genes, was shown to be decreased, consistent with the effect of NO on biofilm observed in the same study (Henares et al., 2013). Interestingly, however, the effect of NO on biofilm and flagellin concentration was NO concentration-dependent, however; as NO concentration increased, biofilm decreased and flagellin proteins increased (50–200 nM NO was studies). Nevertheless, since the loss of flagellin is associated with biofilm formation, these experiments provide a possible mechanism by which low nanomolar NO influences QS-mediated biofilm formation through the H-NOX/HqsK pathway.

Key concept 1: NO mediates biofilm enhancement analogously to a high cell-density state in *Vibrio haveryi*.

### *Vibrio cholerae* Contains a NO Sensing Hybrid Histidine Kinase Receptor

**FIGURE 2:**
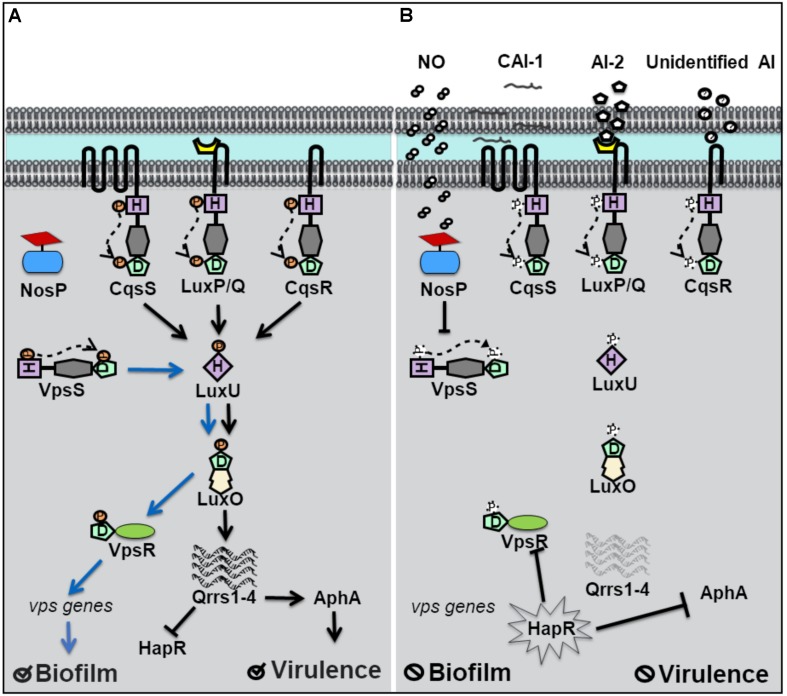
Quorum sensing (QS) in *Vibrio cholerae* at high and low cell density. **(A)** At low cell density, when autoinducer concentration is small, *V. cholerae* hybrid histidine kinase receptors CqsS, LuxPQ, and CqsR transfer phosphate to a histidine phosphotransfer protein, LuxU. In the absence of NO, the cytosolyic hybrid histidine kinase VpsS, acts as a kinase, and participates in phosphotransfer with LuxU. Phosphorylated LuxU tranfers phosphate to the transcription factor LuxU, resulting in the transcription of four small regulatory RNA molecules (Qrrs 1-4). Qrrs activate the master regulator of virulence, AphA, and inhibit the expression of HapR, the master repressor of vibrio polysaccharide (*vps*) gene expression. Low cell density favors biofilm formation and virulence factor secretion. **(B)** QS signal transduction at high cell density and in the presence of NO. When cell density is high, and autoinducer concentrations rise, CqsS, LuxPQ, and CqsR act as phosphatases, dephosphorylating LuxU. Qrrs are not expressed and HapR is transcribed, resulting in the inhibition of AphA and genes involved in *vps* expression. Likewise, in the presence of NO, VcNosP FeII–NO inhibits the autophosphorylation activity of VpsS and results in decreased phosphate flux through LuxU.

*Vibrio cholerae* relies on QS for the regulation of biofilm and virulence related genes (Kovacikova and Skorupski, 2002; Hammer and Bassler, 2003). Vibrio polysaccharide genes (*vps*), involved in the synthesis of the exopolymeric matrix of biofilms, and the master regulator of virulence factor production, AphA, are under QS control. Four separate QS pathways are believed to work in parallel in *V. cholerae* to allow for the detection of multiple signals (Jung et al., 2016). Four hybrid histidine kinase receptors have been identified. Three of these receptors, CqsS, LuxP/Q, and Vc1831 (CqsR), are membrane bound and the fourth, VpsS, is cytosolic (Figure 2; Miller et al., 2002; Shikuma et al., 2009). When autoinducer concentrations are high, CqsS, LuxP/Q and CqsR bind their cognate autoinducers and act as phosphatases, drawing phosphate away from the common phosphotransfer protein LuxU. At high cell density, HapR, the homolog of *V. harveyi*’s LuxR, is expressed, and represses genes responsible for biofilm and the master regulator of virulence factors, AphA (Rutherford et al., 2011). Therefore at high cell density, *V. cholerae* disperses from biofilm and is latent. However, when the concentration of autoinducers is small, typically at low cell density, the membrane bound receptors act as kinases and initiate phosphoryl transfer to LuxU and subsequently to the transcription factor LuxO. As in *V. harveyi*, phosphorylated LuxO in *V. cholerae*, initiates the expression of small regulatory RNA molecules qrrs (1-4) which inhibit HapR activity and also stabilize AphA (Svenningsen et al., 2008; Shao and Bassler, 2012). Repression of HapR activity derepresses expression of AphA and biofilm *vps* genes, resulting in increased virulence and biofilm at low cell density.

Until recently, the cognate autoinducer for the cytosolic kinase receptor VpsS was unknown, though VpsS was predicted to participate in QS based on studies demonstrating its purified receiver domain accepts phosphate from phosphorylated LuxU *in vitro* (Shikuma et al., 2009). Early studies of VpsS found that overexpression of *vpsS* in *V. cholerae* resulted in LuxO-mediated activation of *vps* genes and a hyperbiofilm phenotype. LuxO activation of *vps* genes was found to occur by activation of the positive regulator of *vps* gene expression, VpsR and is independent of HapR. Recently, our lab has shown that full-length VpsS participates in phosphotransfer with LuxU (Hossain et al., 2018). Like HqsK in *V. harveyi*, VpsS does not contain a sensory domain, which suggests that an accessory protein is used to regulate its activity. Our lab discovered that VpsS is co-cistronic with a novel NO sensing hemoprotein called NosP. Further, we showed that purified NO-bound NosP inhibits the autophosphorylation of VpsS, which subsequently results in decreased levels of phosphorylated LuxU *in vitro* (Hossain et al., 2018).

These experiments suggest that *V. cholerae* may sense NO to regulate gene expression through a QS pathway involving LuxU and LuxO. As in *V. harveyi*, NO appears to act analogously to an autoinducer to mimic a high cell density state where phosphate is transferred downstream through LuxU. Interestingly, *V. cholerae* has been shown to disperse from biofilm in the presence of nanomolar NO (Barraud et al., 2009). A decrease in phosphate flux downstream, as a result of NO-bound NosP inhibition of VpsS autophosphorylation, would explain the biofilm dispersal phenotype of *V. cholerae* in the presence of NO, as *vps* genes responsible for biofilm formation would be repressed. The relative effect of NO-contributed phosphate flux compared to that of the three other QS pathways in *V. cholerae* has not been quantified. It is possible, that like with *V. harveyi*, NO modulation of QS occurs predominantly at low cell density and/or is a minor overall contributor to phosphate flux through LuxU/LuxO. It is also possible that NO-modulated QS represents the broader possibility that exogenous environmental signals are able to modulate QS, a departure from QS outputs dependent on only true autoinducing (and thus cell density-dependent) molecules.

## No and Innate Immunity

In response to a bacterial infection, the mammalian host produces high levels of NO through the upregulation of (iNOS). Nitrosative stress is an important component of the hosts innate immunity as it curbs microbial growth through the disruption of the fundamental physiological process including respiration, metabolism, and DNA replication (Murphy, 1999). However, bacteria have evolved to cope with nitrosative stress in order to circumvent host defenses during infection. In this section, we will discuss the discovery of a NO sensitive QS circuit in *Staphylococcus aureus* that may provide the bacterium with an evolutionary advantage when encountering high levels of NO during infection of a human host.

### Nitrosylation of a QS Regulator Inhibits Virulence of *S. aureus*

**FIGURE 3:**
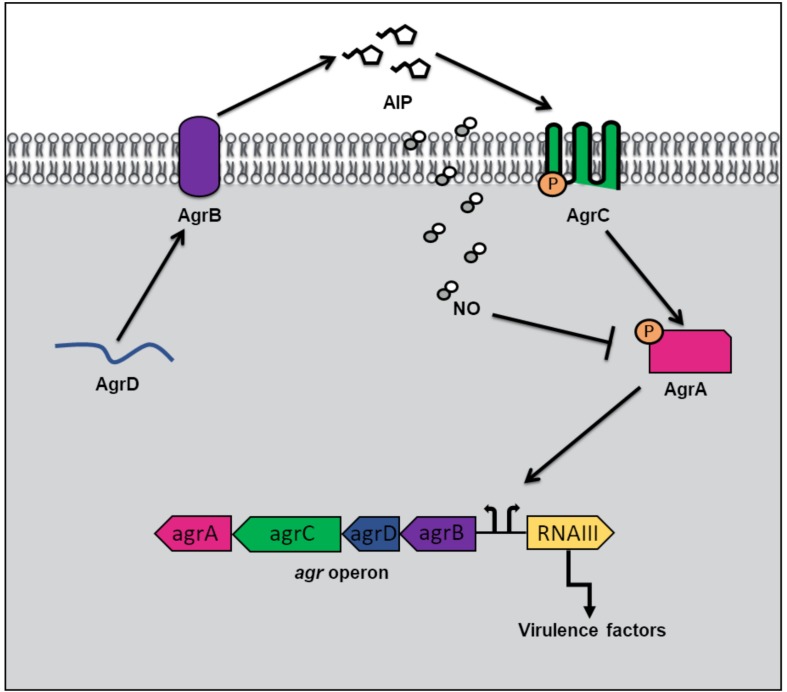
Quorum sensing (QS) in *Staphylococcus aureus* at high cell density and in the presence of nitric oxide (NO). QS in *S. aureus* is mediated by an autoinducing peptide (AIP) that is formed from the cleavage of the propeptide AgrD. AgrD is exported across the membrane by the endopeptidase AgrB. At high cell density, the concentration of AIP rises until a threshold level is reached, and binding to AgrC receptor results. AgrC autophosphorylates upon binding AIP, and subsequently transfers its phosphate to the transcription factor AgrA. Phosphorylated AgrA induces the positive autoregulation of the *agr* operon, as well as the transcription of RNAIII, resulting in the expression of virulence factors. In the presence of NO, AgrA is inhibited, resulting in decreased transcription of the *agr* operon as well as toxin genes.

In addition to hemoprotein coordination, NO may exert its influence over bacterial QS pathways by protein S-nitrosylation (Kovacs and Lindermayr, 2013). NO modification of cystine residues is an important post translation modification governing protein function that has been observed in mammals, bacteria and plants. Free radical NO can react directly with thiyl radicals or may first react with an oxidant such as superoxide, oxygen or redox metals to form S-nitrosylating agents like peroxynitrite (Kovacs and Lindermayr, 2013). Not surprisingly, considering the inhibitory effect of NO on microbial survival, targets of S-nitrosylation within the genome of the commensal bacterium, *S. aureus*, include proteins involved in carbohydrate and lipid metabolism, tRNA and cell wall biosynthesis, DNA replication and repair, and amino acid metabolism (Urbano et al., 2018). Interestingly however, a small subset of S-nitrosylated proteins in S. *aureus* are responsible for antibiotic resistance and virulence. AgrA, a major transcriptional activator of virulence genes and a key component of staphylococcal QS, was found to be a target of S-nitrosylation at three separate cysteine residues C6, C123, and C199 (Urbano et al., 2018). In *S. aureus*, when cell density is high, AgrA is activated through phosphotransfer from the autophosphorylated AgrC receptor (Figure 3). When phosphosphylated, AgrA subsequently binds to several promotors responsible for the expression of virulence factors, including agrPIII, leading to positive autoregulation of the *agr* operon. Upon addition of a NO donor, the transcription of several AgrA targets was inhibited in a concentration dependent matter. Further, a NO insensitive mutant, in which C199 was replaced with serine residue, exhibited resistance to NO (Urbano et al., 2018). These findings suggest that NO inhibits QS-mediated virulence in *S. aureus* through the S-nitrosylation of AgrA cysteine residues, particularly C199. The authors also hypothesize that considering the conservation of cysteine residues across the LytTR family of regulators, NO might affect similar processes in other bacterial species through cysteine modification.

Key concept 2: S-nitrosylation of AgrA cysteine residues impedes binding to target promoters.

α-toxin is a pore forming toxin that is the major contributor of *S. aureus* pathogenesis (Berube and Bubeck Wardenburg, 2013). In *S. aureus* pneumonia, α-toxin is responsible for lung injury and inflammation (Bartlett et al., 2008). The production of α-toxin is regulated by AgrA, as activation of agrPIII induces RNAIII, a regulatory RNA molecule responsible for stimulation of α-toxin transcription (Morfeldt et al., 1995). The Fang laboratory discovered that the QS pathway used to regulate α-toxin production in *S. aureus* is affected by NO (Urbano et al., 2018). Congenic iNOS knockout mice were more susceptible to *S. aureus* infection than iNOS-competent mice suggesting that NO serves a protective role in the host during infection. While there were no significant differences in bacterial burden in the lungs of mice with and without iNOS, significantly higher levels of α-toxin were produced in iNOS-deficient mice compared with iNOS-competent mice (Urbano et al., 2018). Further, iNOS-deficient mice infected with a *S. aureus* mutant, in which C55 and C199 were replaced with NO insensitive residues, exhibited no differences in α-toxin levels compared to iNOS competent mice. Taken together, the *in vitro* and *in vivo* findings provide a mechanism by which NO serves a protective role during *S. aureus* infection by inhibiting AgrA activation of toxin expression through S-nitrosylation of AgrA cysteines required for promotor binding. The authors speculate that a potential advantage to *S. aureus*, of having a NO sensitive QS circuit to represses virulence, may be to maintain a balance between a pathogenic and commensal lifestyle during colonization of the human nose. Interestingly, asymptomatic nose carriers of *S. aureus* were found to contain bacteria with weak expression of Agr regulated toxins (Burian et al., 2010).

Key concept 3: In a *S. aureus* model of infection, virulence, and not bacterial burden, is responsible for the differential disease severity between iNOS-deficient and iNOS-competent mice.

## Concluding Remarks

In this review, the influence of NO on the QS-regulated behaviors of *V. harveyi*, *V. cholerae*, and *S. aureus* have been discussed. In *Vibrios*, NO ligation to hemoproteins has been found to influence the autophosphorylation of partner histidine kinases proteins that are integrated in QS pathways. NO appears to influence QS-mediated behaviors via a different mechanism in *S. aureus*, namely S-nitrosylation of a QS regulator protein. It has yet to be determined whether S-nitrosylation occurs in other bacterial species (including *V. harveyi* and *V. cholerae*) as a means to regulate cell-to-cell communication. Based on the currently available data, it is likely that additional, as of yet undiscovered, NO-sensitive QS pathways exist in other bacterial species, both Gram-negative and Gram-positive strains. Like the examples first characterized and described here, these pathways are likely to detect NO concentrations through ligation to a hemoprotein or S-nitrosylation of cysteine residues.

Major questions concerning the role of NO in QS are outstanding and should be the subject of future study. There is no current evidence that NO is truly an autoinducer, as it may not be self-synthesized. Thus, one major question for future studies is what is the source of NO detected by QS circuits? Furthermore, if exogenous (either environmental or eukaryotic) NO is informing bacterial group decision making, another major open question is why? Finally, as molecules alternative to traditional AIs, including NO and lipids, have now been shown to modulate QS pathways, thus it may be appropriate to expand our understanding of QS from the traditionally understood role of monitoring cell density.

## Author Contributions

IH and EB wrote the manuscript.

## Conflict of Interest

The authors declare that the research was conducted in the absence of any commercial or financial relationships that could be construed as a potential conflict of interest.
